# Bovine Hemoglobin Enzymatic Hydrolysis by a New Ecoefficient Process—Part I: Feasibility of Electrodialysis with Bipolar Membrane and Production of Neokyotorphin (α137-141)

**DOI:** 10.3390/membranes10100257

**Published:** 2020-09-25

**Authors:** Mira Abou-Diab, Jacinthe Thibodeau, Barbara Deracinois, Christophe Flahaut, Ismail Fliss, Pascal Dhulster, Naima Nedjar, Laurent Bazinet

**Affiliations:** 1 Department of Food Science, Université Laval, Quebec, QC G1V0A6, Canada; mira.abou-diab.1@ulaval.ca (M.A.-D.); jacinthe.thibodeau.1@ulaval.ca (J.T.); ismail.fliss@fsaa.ulaval.ca (I.F.); 2 Laboratory of Food Processing and Electromembrane Process (LTAPEM), Université Laval, Quebec, QC G1V0A6, Canada; 3 Institute of Nutrition and Functional Foods (INAF), Université Laval, Quebec, QC G1V0A6, Canada; 4 UMR Transfrontalière BioEcoAgro N°1158, Université Lille, INRAE, Université Liège, UPJV, YNCREA, Université Artois, Université Littoral Côte d’Opale, ICV—Institut Charles Viollette, F-59000 Lille, France; barbara.deracinois@univ-lille.fr (B.D.); christophe.flahaut@univ-artois.fr (C.F.); pascal.dhulster@univ-lille.fr (P.D.)

**Keywords:** bovine hemoglobin, electrodialysis with bipolar membrane, enzymatic hydrolysis, neokyotorphin, peptides, membrane fouling

## Abstract

Neokyotorphin (α137-141) is recognized as an antimicrobial peptide and a natural meat preservative. It is produced by conventional enzymatic hydrolysis of bovine hemoglobin, a major component of cruor, a by-product of slaughterhouses. However, during conventional hydrolysis, chemical agents are necessary to adjust and regulate the pH of the protein solution and the mineral salt content of the final hydrolysate is consequently high. To produce this peptide of interest without chemical agents and with a low salt concentration, electrodialysis with bipolar membrane (EDBM), an electromembrane process recognized as a green process, with two different membrane configurations (cationic (MCP) and anionic (AEM) membranes) was investigated. Hydrolysis in EDBM showed the same enzymatic mechanism, “Zipper”, and allowed the generation of α137-141 in the same concentration as observed in conventional hydrolysis (control). EDBM-MCP allowed the production of hydrolysates containing a low concentration of mineral salts but with fouling formation on MCP, while EDBM-AEM allowed the production of hydrolysates without fouling but with a similar salt concentration than the control. To the best of our knowledge, this was the first time that EDBM was demonstrated as a feasible and innovative technology to produce peptide hydrolysates from enzymatic hydrolysis.

## 1. Introduction

Blood from slaughterhouses is an inevitable part of the meat production but also a natural bioresource produced in very large quantities. Its current use is mainly limited to the animal food chain (controversial concept in the context of prevention of animal diseases) or intended for landfills. Hence, only 30% of the blood is valorized [1], while it has a high protein content and is an ideal substrate for proteolysis. For this reason, in the context of the circular economy, of which the objective is to produce goods while strongly limiting the consumption of raw materials and non-renewable energy sources, the agri-food industry is turning today towards the recovery of this waste and seeking to develop products with high added value.

After centrifugation, the slaughter cattle blood is essentially subdivided into two parts. The colorless part, plasma, equivalent to around 60% of the volume of blood, can be used in pharmaceuticals due to its high content of thrombin, fibrinogen or albumin serum [2]. The second part, cruor, responsible for the red color of the blood, is considered as waste and represents up to 40% of the blood volume. However, cruor contains mainly hemoglobin (90%). Bovine hemoglobin is a 64,500 Da protein, consisting of four polypeptide chains, two α chains and two β chains [3]. The hydrolysis of bovine hemoglobin with pepsin is a source of many peptides with biological activities such as opioid [4,5,6], hematopoietic [7] and antihypertensive [8]. Nevertheless, the most described aspect remains the antimicrobial activity [9,10,11,12]. One of these antimicrobial peptides, the α137-141 peptide (neokyotorphin, 653 Da, pI of 10.5), has recently been reported as a natural preservative to protect meat during its storage or distribution. α137-141 showed a 60% reduction in the rancidity time and inhibited microbial growth after 14 days in the refrigerator with effects close to butylhydroxytoluene (BHT), commonly used as a meat preservative [12].

The most commonly used procedure to generate the α137-141 peptide from hemoglobin is conventional enzymatic hydrolysis [13,14,15]. It includes several steps consisting of hemoglobin denaturation by chemical acidification, hydrolysis with pepsin enzyme and pH adjustment and inactivation of enzymatic reaction by chemical basification. However, the hydrolysates produced contain high levels of mineral salts due to the addition of chemicals. Therefore, in this study, it is proposed to apply a green technology [16], named electrodialysis with bipolar membrane (EDBM), as an alternative method to the conventional enzymatic hydrolysis of hemoglobin to obtain α137-141. EDBM consists in combining homopolar membranes with bipolar membranes, whose membranes generate H^+^ ions from water dissociation under an electrical field inducing acidification of the solution at the cationic interface of the bipolar membrane (BM). One major advantage of EDBM reported by Bazinet et al. [17] is the possibility to acidify and demineralize a stream simultaneously, so that the electro-acidified product has higher purity than a chemically acidified product. EDBM technology was previously used for solubilization of chitosan before its enzymatic hydrolysis using bipolar membrane electroacidification [18]. Recently, EDBM was applied to produce deacidified cranberry juice, with a drastic energy consumption reduction and ecoefficiency improvement [19]. Consequently, by using EDBM, the whole traditional process necessary for the production of peptide α137-141 from hemoglobin in a single technology without the addition of acids, to acidify the protein solution before its hydrolysis and subsequently to regulate the pH of the reaction medium, would be simplified and more sustainable. Eventually, the hydrolysates produced would contain low levels of mineral salts since no acid would be added and the solution would be demineralized. However, EDBM has never been tested for hemoglobin hydrolysis and the optimal configuration has yet to be determined. 

In this context, the present study aimed to prove the feasibility of enzymatic hydrolysis of bovine hemoglobin by EDBM to obtain α137-141. The specific objectives of this study were to (1) study the impact of stacking anion or cation-exchange membranes as homopolar membranes in EDBM on pH and conductivity evolution, (2) identify the enzymatic mechanism involved in EDBM hydrolysis, (3) characterize the peptide population obtained after hydrolysis, (4) quantity the production of α137-141 and finally, (5) compare the efficiency of EDBM processes with the conventional hydrolysis.

## 2. Materials and Methods 

### 2.1. Materials

Hemoglobin. The purified hemoglobin powder from bovine blood, dark brown, was purchased from Sigma-Aldrich (H2625, Oakville, ON, Canada). Its composition is given in Table 1. Hemoglobin was stored at 4 °C before use.

Pepsin. The pepsin lyophilized powder from porcine gastric mucosa was purchased from Sigma-Aldrich (P6887, Oakville, ON, Canada). The activity of pepsin was measured at 3250 AU/mg of protein according to a protocol established by the supplier Sigma-Aldrich (Saint-Quentin-Fallavier, France). Pepsin was stored at −20 °C. 

### 2.2. Electrodialysis Cell Configuration

**Figure 1:**
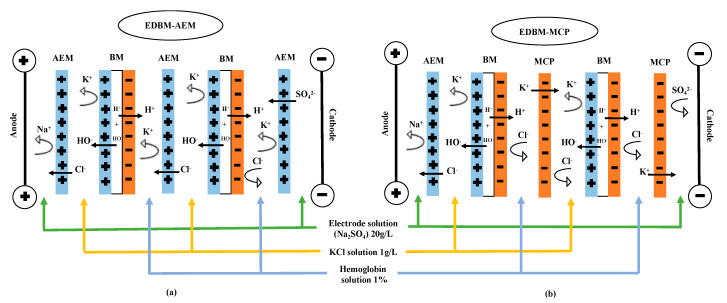
Schematic representation of electrodialysis cell configurations used for electro-acidification and hydrolysis of bovine hemoglobin in (**a**) electrodialysis with bipolar and anion exchange membranes (EDBM-AEM) and (**b**) electrodialysis with bipolar and monovalent cation permselective membranes (EDBM-MCP). MCP: monovalent cation permselective membrane; AEM: anion exchange membrane; BM: bipolar membrane.

The electrodialysis (ED) experiments were carried out using a Microflow-type cell (10 cm^2^ of effective membrane surface). The anode was a plate dimensionally stable electrode (DSA-O_2_, Ti/IrO_2_ coating) and the cathode a 316-stainless-steel electrode from ElectroCell AB (Taby, Sweden). Two ED configurations were tested: the ED configuration named “EDBM-AEM” (Figure 1a) formed by stacking three anion-exchange membranes (AEM, Astom, Tokyo, Japan) and two bipolar membranes (BM, Astom, Tokyo, Japan) and the ED configuration named “EDBM-MCP” (Figure 1b) stacking one anion-exchange membrane (AEM, Astom, Tokyo, Japan), two monovalent cation permselective membranes (MCP, Japan Food grade Neosepta, Astom, Tokyo, Japan) and two bipolar membranes (BM, Astom, Tokyo, Japan) (Figure 1b). Monovalent ion permselective membranes are a category of ion-exchange membranes allowing the migration of oppositely charged monovalent ions while retaining the polyvalent ions. Monovalent cation permselective (MCP) membranes are made of resin, including negative charges coated with a thin positive charge layer [20,21]. Such layers on both membrane interfaces are able to separate ions of the same charge but with different valences. With this particular layer on its interface, the MCP may increase its resistance to peptide fouling, as demonstrated recently by Persico et al. [22]. This was the reason for choosing this type of membrane for our study. In both cases, the ED configuration defined three closed loops containing 200 mL of hemoglobin solution (1% Hb w/v), 200 mL of salt ion recovery (KCl 1 g/L (EMD Chemicals Inc, Port Wentworth, GA, USA)) and 250 mL of electrode-rinsing solution (Na_2_SO_4_ 20 g/L (ACP Inc., Montréal, QC, Canada)).

### 2.3. Protocol 

To assess the feasibility of EDBM for enzymatic hydrolysis of bovine hemoglobin by pepsin, three different conditions were tested: conventional hydrolysis in beaker, hydrolysis by EDBM-MCP and hydrolysis by EDBM-AEM. The conventional hydrolysis in beaker was considered as the “control” since it has been demonstrated in several enzymatic hydrolysis studies to allow the production of α137-141 peptide with various biological activities [10,12,23,24]. Hence, all results were compared to this control.

#### 2.3.1. Conventional Hydrolysis 

##### Stock Solution Preparation

A stock solution was prepared by adding 15 g of purified bovine hemoglobin (BH) to 100 mL of ultrapure water. After centrifugation (6000× *g* for 30 min (Eppendorf AG, 22331 Hamburg, Germany; Centrifuge 5804 R, Brinkmann Instruments, Westbury, NY, USA)), the supernatant was recovered and the real BH concentration (C_BH_) was determined according to Drabkin’s method [25]. From the concentration C of the stock solution, a solution of purified BH was prepared by dilution to a 1% (w/v) precise concentration of hemoglobin C_BH_. The pH of the 1% hemoglobin solution was 6.94 ± 0.1 and its conductivity value 58.36 ± 1.33 μS/cm. 

##### Hydrolysis Process

Since the stock solution was composed of hemoglobin in its native form, the pH was first adjusted to 3 with HCl (2M, Anachemia, VWR Company International, Mississauga, ON, Canada) to denature hemoglobin. Then, the hydrolysis was carried out at pH 3 in the presence of porcine pepsin (Sigma-Aldrich, Oakville, ON, Canada) from porcine gastric mucosa (EC 3.4.23.1, 3200–4500 units mg^−1^ protein) solubilized in ultrapure water, with an enzyme/substrate ratio equal to 1/11 (mole/mole). Pepsin is an enzymatic protein belonging to the aspartyl proteases family [26]. This endopeptidase catalyzes the hydrolytic cleavage of peptic hydrophobic or aromatic amino acid residues. 

#### 2.3.2. Hydrolysis by EDBM-MCP and EDBM-AEM

The 1% hemoglobin solution was prepared as previously, except that (1) the conductivity was adjusted to 1.5 mS/cm with KCl addition since the 1% HB solution conductivity was too low for ED experiments and (2) the pH was adjusted to 3 by applying an electric current, leading to the electrochemical production of protons H^+^ at the cationic interface of the bipolar membrane in contact with the HB solution. When the pH value of 3 was reached, porcine pepsin was added to start the hydrolysis. The protocol for both conventional and EDBM hydrolysis is presented in the Supplementary Materials, Figure S1. 

The flow rate of the electrode-rinsing solution was set at 450 mL/min and the flow rates of 1% HB and KCl solutions were set at 400 mL/min. Each closed loop was connected to a separate external reservoir, allowing a continuous recirculation of each compartment (Figure 1). A constant voltage of 15 V, corresponding at the beginning of the process to a current density of 5.3 mA/cm^2^, was applied during the EDBM experiments using a BK Precision power supply (Model BK9110-ND, Vancouver, BC, Canada). During each experiment, the hydrolysis was conducted for 3 h at 30 °C. Samples were taken at 0 min before adding the pepsin and then after pepsin addition at 2.5, 30, 60, 120 and 180 min during the hydrolysis step, corresponding to different degrees of hydrolysis. The reaction in each sample was stopped by addition of KOH (0.5M) (Fisher Scientific, Branchburg, NJ, USA) in order to bring the sample to pH 9, allowing both to irreversibly inactivate the enzyme and to avoid any possible basic hydrolysis. The samples were stored at −20 °C until further analyses. The current intensity, conductivities, pH and temperature, for both 1% HB and KCl solutions, were recorded every 30 min during EDBM treatments. Three randomized repetitions of each EDBM and control condition were carried out. For EDBM, after each treatment, a cleaning in place (CIP) was performed according to the manufacturer’s procedure, the cell was dismantled and the membrane conductivity and thickness measured to evaluate their integrities. The membranes were changed for new membranes after each repetition. Hydrolysis mechanism and peptide identification were carried out on samples collected during hydrolysis (T_0_, T_2.5min_, T_30min_, T_60min_, T_120min_ and T_180min_).

### 2.4. Analyses

#### 2.4.1. Determination of Hemoglobin Concentration: Drabkin’s Method

Drabkin’s reagent is a spectrophotometric method used for the quantitative determination of hemoglobin concentration in whole blood. Briefly, 40 µL of the sample were added to 10 mL of the Drabkin’s reagent (D5941, Sigma-Aldrich, Oakville, ON, Canada) for 15 min at room temperature. Then, the absorbance was read at a wavelength of 540 nm by a UV spectrophotometer (ChemStation 8453A UV-Vis Spectrophotometer, Agilent Technologies, Santa Clara, CA, USA) and reported into the calibration curve. From the concentration C of the stock solution, solution of purified bovine hemoglobin was prepared by dilution to precise concentrations of hemoglobin C_BH_: 1% (w/v). 

#### 2.4.2. pH

The pH values of the 1% HB and KCl solutions were measured with a pH meter (model SP70, symphony, VWR international, Montreal, QC, Canada) equipped with an automatic temperature compensation (ATC) pH probe. To stop the hydrolysis reaction in the hemoglobin hydrolysate samples, the pH was measured with a fine tip pH-electrode (Mettler Toledo, LE422, micro, 3 mm, Leicester, UK). 

#### 2.4.3. Conductivity

Conductivity of the 1% HB and KCl solutions was measured with a YSI conductivity meter (Model 3100, Yellow Springs Instrument, Yellow Springs, OH, USA) combined with a YSI 3252 electrode with a cell constant of 1 cm^−1^. 

#### 2.4.4. EDBM Global Resistance 

The global system resistance was calculated according to Ohm’s law: R = U/I, where R is the global system resistance (Ω), I the current intensity (A) and U the voltage applied between the electrodes of the ED cell (V). The voltage and the current intensity were directly read on the power supply. 

#### 2.4.5. Membrane Thickness and Electrical Conductivity

Membrane thickness was measured with an electronic digital micrometer (Model Marathon Watch Company LTD., Richmond Hill, ON, Canada).

Membrane electrical conductance (G) was measured with a specially designed cell (Laboratoire des Matériaux Échangeurs d’Ions, Créteil, France) following the procedure described by Mikhaylin et al. [27]. The NaCl reference solution was 0.5 M. The membrane conductivity was calculated according to Lteif et al. [28] and Lebrun et al. [29] (Equation (1)):

R_m_ = R_m+s_ − R_s_ (1)

where R_m_ is the electrical resistance of the membrane (in Ω), R_m+s_ the electrical resistance of the membrane and the reference solution measured together (in Ω) and R_s_ the electrical resistance of the reference solution (in Ω). The resistance R is equal to 1/G. The membrane electrical conductivity (κ) was calculated according to Equation (2):

κ = L/R_m_A (2)

where L is the membrane thickness (in cm) and A is the electrode area (1 cm^2^). These measurements were recorded before and after each replicate.

#### 2.4.6. Total Solid and Ash Contents

The total solid content was determined by drying hemoglobin solution samples at 95 °C overnight. The ash content was measured by heating the dried samples at 550 °C for 20 h according to the AOAC international method 945.46 [30].

#### 2.4.7. Mineral Concentration Measurement

The samples collected after 180 min of hydrolysis were thawed at 4 °C before their dilution 1/20 in Milli-Q water. Potassium, sodium and sulfur concentrations were determined by optical emission spectrometry with inductively coupled plasma as atomization and excitation source (ICP-OES Agilent 5110 SVDV Agilent Technologies, Victoria, Australia), using the following wavelengths (in nm): 766.491 (K), 588.995; 589.592 (Na), 181.972; 180.669 (S). The analyses for all ions were carried out in axial and/or radial view. For chlorides, the samples were analyzed with Flow Injection Analysis (FIA) (Quikchem 8500 series 2, Zellweger Analytic, inc., Lachat Instruments Division, Milwaukee, WI, USA) with Quikchem method 10-117-07-1-C: Determination of chloride by Flow Injection Analysis colorimetry. 

#### 2.4.8. Determination of the Degree of Hydrolysis of Bovine Hemoglobin

The degree of hydrolysis (DH) is defined as the ratio between the number of peptide bonds cleaved by an enzyme and the number of total substrate bonds (protein), according to Equation (3):

(3) $$\mathrm{DH}=100\times \frac{h}{h_{0}}$$

where DH is the degree of hydrolysis (%), h is the number of peptide bonds hydrolyzed in the substrate and $h_{0}$ is the number of total peptide bonds of the protein substrate.

DH was quantified by the ortho-phthaldialdehyde (OPA) technique, adapted from Church et al. [31] and Spellman et al. [32]. In order to determine the number h of hydrolyzed bonds, Equation (4) was used:

(4) $$h=\frac{\Delta \mathrm{Abs}\cdot M\cdot \mathrm{DF}}{\varepsilon \cdot C_{\mathrm{HB}}}$$

where ΔAbs is the difference between the absorbance at 340 nm of the hydrolyzed sample and the absorbance of the unhydrolyzed sample, M the molar mass of the protein (for bovine hemoglobin: 64,500 g·mol^−1^), DF the dilution factor of the sample, ε the molar extinction coefficient of OPA reagent at 340 nm (6000 mol^−1^·cm^−1^ according to Church et al. [33]) and $C_{\mathrm{HB}}$ the concentration of bovine hemoglobin (10 g/L equivalent to 1%). 

The reagent was prepared at least one hour before use and stored in the dark. For a final volume adjusted to 50 mL, its composition was 25 mL of sodium tetraborate solution (0.1M) (Fisher Chemicals, Belgium), 5 mL of 10% (w/v) SDS (Fisher Scientific, Branchburg, NJ, USA), 10 μL of β-mercaptoethanol (Sigma-Aldrich, Oakville, ON, Canada) and 80 mg of OPA (Sigma-Aldrich, Oakville, ON, Canada) dissolved in 1 mL of methanol (Fisher Chemicals, Belgium). The linearity of the reagent was verified by assaying L-leucine (Sigma-Aldrich, Oakville, ON, Canada). A volume of 50 μL of sample to be tested was added to 1 mL of reagent. The absorbance was read at 340 nm after incubation for 2 min at room temperature. 

#### 2.4.9. RP-UPLC Analyses 

##### Identification of Hydrolysis Mechanism and α137-141 Peptide

Reversed phase ultra-performance liquid chromatography (RP-UPLC) analyses were performed on all samples used a 1290 Infinity II UPLC (Agilent Technologies, Santa Clara, CA, USA). The RP-UPLC apparatus was composed of a binary pump (G7120A), a multisampler (G7167B), an in-line degasser and a variable wavelength detector (VWD G7114B) adjusted to 214 nm. Then, 0.22 µm polyvinylidene difluoride (PVDF) filters were used to filter hydrolysis fractions into a glass vial. Samples were loaded (0.25 µL) into a Poroshell 120 EC-C18 column (2.1 × 100 mm i.d., 2.7 micron, Agilent, Santa Clara, CA, USA). A flow rate of 500 µL/min at 23 °C was used to operate the column. A gradient was applied for the mixture of solvent A (LC-MS grade water with 0.1% formic acid) and solvent B (LC-MS grade ACN with 0.1% formic acid) with solvent B increasing from 1% to 13% in 6 min, to 35% until 25 min and to 100% until 35 min and holding until 45 min, then back to initial conditions. Each sample analysis was performed in triplicate for ensuring technical reproducibility.

To identify and quantify the relative abundances of the peptides present in the samples, a hybrid ion mobility quadrupole time of flight mass spectrometer 6560, IM-Q-TOF, Agilent, Santa Clara, CA, USA) was used. All LC-MS/MS experiments were acquired using Q-TOF. A positive mode at Extended Dynamic Range, 2 Ghz, 3200 m/z with a scan range of 100–3200 m/z was used to record the MS/MS signals. The drying gas was nitrogen at 13.0 L/min and 150 °C and was also used as nebulizer gas at 30 psig. The capillary voltage was set at 3500 V while the nozzle voltage at 300 V and the one of the fragmentors at 400 V. The instrument calibration was carried out by using an ESI-L low concentration tuning mix (G1969-85000, Agilent Technologies, Santa Clara, CA, USA). Data acquisition and analysis were performed with the Agilent Mass Hunter Software package (LC/MS Data Acquisition, Version B.08.00 and Qualitative Analysis for IM-MS, Version B.07.00 Service Pack 2 with BioConfirm Software, Santa Clara, CA, USA).

UPLC-MS/MS coupling was used to analyze the α137-141 peptide. This coupling offered the advantage of a complementarity between UV profile from the UPLC and the masses of the corresponding compounds for each point of the chromatogram. This also gives the amino acid composition of each compound. Several parameters were taken into account to ensure good identification of the α137-141 peptide: the retention time, the monoisotopic molecular mass and the second-order derivative spectral analysis of peptides testified of the presence (or absence) of aromatic amino acids [34]. The α137-141 peptide was identified by comparing it with alpha hemoglobin sequence for bovine hemoglobin hydrolysis by pepsin. 

##### Identification and Characterization of Peptide Population

After sample drying, peptides were dissolved in H_2_O/0.1% TFA at 0.5 mg/mL and centrifuged for 5 min at 10,000× *g*. Then, 10 µL was separated by chromatography at 30 °C on an ACQUITY UPLC system (Waters Corporation, Guyancourt, France) equipped with a Kinetex 2.6 µm C18 100 Å (150 mm × 4.6 mm) column (Phenomenex). Acetonitrile gradient was set at 0.5 mL/min (the mobile phases were composed of solvent A (0.1% (v/v) formic acid/99.9% (v/v) water) and solvent B (0.1% (v/v) formic acid/99.9% (v/v) acetonitrile): from 5% to 15% solvent B during the first 30 min, from 15% to 30% over 60 min, from 30% to 50% over 10 min and then maintained for 10 min at 95% solvent B. The eluate was then directed into the electrospray ionization source of the qTOF Synapt G2-Si™ (Waters Corporation). The analysis was performed in sensitivity, positive ion and data-dependent analysis (DDA) modes. The source temperature was at 150 °C and the capillary and cone voltages were fixed, respectively, at 3000 and 60 V. Data for m/z values were collected in the range of 100 to 2000 Da with a scan time of 0.2 s. A maximum of 10 precursor ions were chosen for MS/MS analysis with an intensity threshold of 100,000. A collision-induced dissociation (CID) fragmentation mode and a scan time of 0.1 s were chosen for MS/MS data collection.

PEAKS Studio (version 8.5, Bioinformatics Solutions Inc., Waterloo, ON, Canada) software and its database search tools were used for peptide identification. The UniProt database restricted to Bos Taurus was used. A mass tolerance of 35 ppm, 3 missing cleavage sites and an MS/MS tolerance of 0.2 Da were allowed while a variable methionine oxidation was considered. Protein and peptide identity relevance was judged according to their score in the research software (*p* value of 0.05 (*p* < 0.05), False Discovery Rate < 1%). For the search of peptides from the pepsic hydrolysate by Peaks^®^ software, the Bos taurus bovine hemoglobin alpha and beta chain sequences were taken as model proteins. In addition, heat maps were generated to visualize peptide abundance. The results were expressed using a color code where high, low and no occurrence of an individual amino acid were represented in red, yellow and white, respectively.

Progenesis QI for proteomics (Version 4.0, Nonlinear Dynamics, Newcastle upon Tyne, England) was also used to analyze mass spectrometry data. These data were subjected to the following successive processing steps: (i) alignment of the peptide maps with a quality control corresponding to an equivolume mixture of each of the analyzed samples, (ii) peak picking, detection of isotopic massif passing the software intensity threshold, (iii) data standardization to carry out a statistical principal component analysis (PCA). The variables used were derived from the comparison of peptide maps, i.e., the position of the isotopic massifs and their intensity. 

##### Quantification of α137-141 in the Hydrolysates by Mass Spectrometry 

A α137-141 standard was weighed out and dissolved in H_2_O to prepare stock solution at 5 mg/mL. The solution was centrifuged for 5 min at 10,000× *g*. A standard curve at concentrations ranging between 0.40 and 12.5 µg/mL was established by injection of the α137-141 standard. Dried hydrolysates were dissolved at 0.5 mg/mL in H_2_O. The concentration of α137-141 in the samples was determined by the same chromatographic procedure and equipment as previously for the characterization of the sample peptide population. The mobile phases were 0.1% (v/v) formic acid/99.9% (v/v) water (solvent A) and 0.1% (v/v) formic acid/99.9% (v/v) acetonitrile (solvent B). The acetonitrile gradient was set at 0.5 mL/min: 1% solvent B for 1.50 min, from 1% to 20% solvent B over 8.50 min, from 20% to 50% solvent B over 1 min followed by a washing step and an equilibrating procedure, respectively, with 95% and 1% solvent B for 2 min. The eluate was directed as previously and the MS analysis performed in sensitivity, with positive ions. As previously for the identification and characterization of the peptide population, the source temperature, capillary, cone voltages m/z value range and scan time of 0.2 s were set as the same values.

MassLynx V4.1 software (Waters Corporation) was used to analyze the LC-MS data and, in these conditions, the retention time for α137-141 was 3.20 min. Extracted chromatograms from α137-141 ((M + H_2_)^2+^ 327.7) were generated at a mass precision of 0.1 Da. After integration, quantification of α137-141 was performed according to peak areas after validation of isotopic clusters and elution time. To establish a linear relation between the α137-141 concentration in the sample and the area surface from each spectrum, the areas under the mass peak were used, for any hydrolysate, by using Equation (5) (R^2^ = 0.999):

A_α137-141_ = 5935612.61 × C_α137-141_ − 1042.65 (5)

where C_α137-141_ is the α137-141 concentration (μg/mL) and A_α137-141_ is the area peak.

#### 2.4.10. RP-UPLC Statistical Analyses 

All analyses were performed in triplicate and three independent repetitions were carried out for each condition. Data were subjected to one-way or two-way analyses of variance (ANOVA). Tukey tests were also performed on data using SigmaPlot software (Version 14.0, Systat Software, San Jose, CA, USA) to determine which treatment was statistically different from the others at a probability level *p* of 0.05. 

## 3. Results and Discussion 

To test the feasibility of EDBM, to understand the different results obtained and to compare them with the control, the section below will present the parameters of the three conditions: conventional hydrolysis without membranes (control), hydrolysis in electrodialysis with bipolar and cationic membranes configuration (EDBM-MCP) and hydrolysis in electrodialysis with bipolar and anionic membranes configuration (EDBM-AEM). The EDBM hydrolysis reaction is due to the characteristics of the bipolar membranes allowing the generation of the necessary ions without the use of chemical solvents.

### 3.1. Conventional Hydrolysis (Control): Evolution of pH and Conductivity 

**Figure 2:**
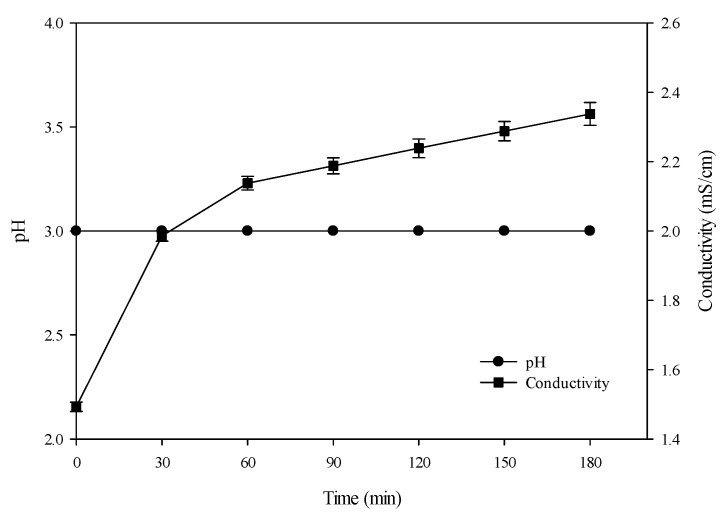
Evolution of pH and conductivity during conventional hydrolysis (control).

To denature hemoglobin, a solution of HCl was added to decrease the pH from 6.81 ± 0.01 to 3. In parallel, conductivity increased from 0.058 ± 0.001 to 1.49 ± 0.013 mS/cm. Once the pH was adjusted to 3, pepsin was added. The hydrolysis phase is shown in Figure 2. Throughout the 3 h of hydrolysis, the pH was maintained at 3 by adding HCl if needed. In parallel, conductivity continued to increase, to reach 2.33 ± 0.032 mS/cm at 180 min. The increase in conductivity was representative of the addition of salts to the solution to adjust the pH.

### 3.2. Hydrolysis in EDBM: Evolution of pH, Conductivity, Global Resistance and Membrane Characterization

#### 3.2.1. pH 

**Figure 3:**
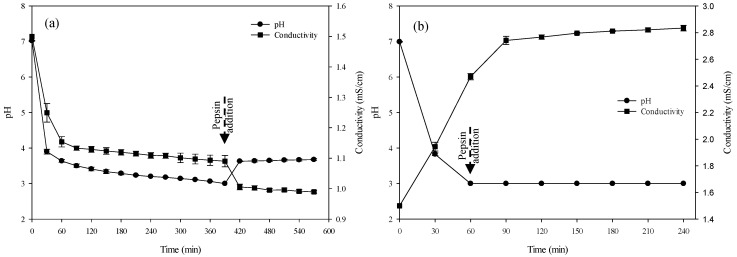
Evolution of pH and conductivity as a function of time during electro-acidification and hydrolysis in EDBM-MCP (**a**) and in EDBM-AEM (**b**).

Hemoglobin electro-acidification process was carried out to decrease the solution pH from 7.02 ± 0.01 to 3 by bipolar membranes which generate the H^+^ ions from water dissociation needed for pH decrease. The mean times required to electro-acidify the 1% HB solution were 390 ± 20 min and 60 ± 2 min in EDBM-MCP and EDBM-AEM, respectively, while the global process durations (electro-acidification + hydrolysis) were 570 ± 20 min and 240 ± 2 min in EDBM-MCP and EDBM-AEM, respectively (Figure 3a,b). The shorter duration of the acidification step observed in EDBM-AEM was explained by the fact that H^+^ ions generated by the bipolar membrane were retained in the hemoglobin compartment by the AEM, not allowing the migration of the H^+^ due to the similar charges of its positive fixed groups (N^+^(CH_3_)_3_, as described by Persico et al. [35] repulsing the H^+^ by Donnan’s effect. Consequently, the H^+^ ions generated were globally all used to decrease the pH of the solution. However, in EDBM-MCP, two phenomena appeared as a function of time. During the first part of the process, the 1% HB solution was depleted in K^+^ migrating through the MCP to the base recovery compartment to form KOH. K^+^ ions were able to cross the cationic membrane due to the opposite charges of its negative fixed groups (SO_3_^−^, as described by Persico et al. [36]) allowing their transfer; the migration of K^+^ ions ensures the electroneutrality of the solution since H^+^ ions were generated in the solution. As observed previously by Bazinet et al. [37] for a two-compartment EDBM process during electroacidification of milk, and Lin Teng Shee et al. [18] for a two-compartment EDBM process during chitosan solubilization by acidification, a decrease in electrical efficiency was observed due to a loss of electrogenerated H^+^ through the cationic membrane, linked to a lack of sufficiently mobile ions such as potassium. Hence, in our case, once the main part of the K^+^ present in the 1% HB solution was migrated, there was a lack of available cations to transport the current and ensure the electroneutrality. Then, the H^+^ has to migrate through the MCP to compensate for this lack of available positively charged species. Those H^+^ ions migrating did not participate anymore in the decrease in pH and then delayed reaching pH 3. During the hydrolysis phase, after adding the solubilized pepsin in water, pH increased normally, as shown in Figure 3. This increase was also observed in the control, due to the change in the medium caused by the chemical reaction between the enzyme and the substrate releasing new molecules. Furthermore, it is important to note that, during the hydrolysis phase in EDBM-MCP, it was more difficult to maintain the pH at 3. Despite the fact that the current remained on throughout the hydrolysis phase, the value remained constant around 3.67 ± 0.03 (Figure 3a). While it was easy to reduce and maintain the pH in EDBM-AEM, the current was applied for a total of 30 min over the 3 h of hydrolysis to maintain the pH constant at 3.0. The difficulty of maintaining the pH at 3.0 in EDBM-MCP can be explained by the lower proton gradient in the hemoglobin compartment in EDBM-MCP than in EDBM-AEM, making difficult the protonation of HB as described for lower pH values in the chitosan compartment with AEM configuration than CEM by Lin Teng Shee et al. [18], and also the difficulty of maintaining the pH can be explained by possible fouling of MCP or BM. 

#### 3.2.2. Conductivity

During EDBM-MCP, conductivity dropped from 1.5 to 0.98 ± 0.004 mS/cm (Figure 3a), which corresponds to a 34% demineralization of the 1% HB solution, while in EDBM-AEM, conductivity increased from 1.5 to 2.83 ± 0.02 mS/cm, corresponding to a mineralization rate of 88% (Figure 3b). The statistical analysis demonstrated that the values of conductivity were significantly different (*p* < 0.05) at the end of the global process of hydrolysis between control (2.33 ± 0.032 mS/cm), EDBM-MCP (0.98 ± 0.004 mS/cm) and EDBM-AEM (2.83 ± 0.02 mS/cm). Conductivity decreased for EDBM-MCP configuration due to the partial demineralization caused by the migration of K^+^ through the MCP [17,38] and increased for EDBM-AEM configuration due to the partial mineralization caused by migration of Cl^−^ anions through the AEM from the basified KCl solution [18,39].

#### 3.2.3. ED System Global Resistance

**Figure 4:**
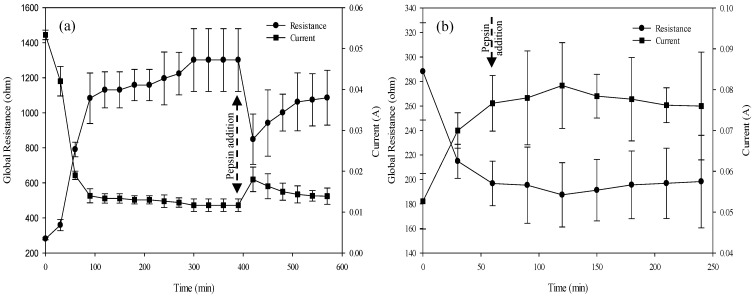
Evolution of the system global resistance and current as a function of time during electrodialysis process in EDBM-MCP (**a**) and in EDBM-AEM (**b**).

At a constant voltage, the current varried throughout the process and was inversely proportional to global resistance (Figure 4). In EDBM-MCP, during the electro-acidification phase, the global resistance increased rapidly from 281.33 ± 6.16 Ω to 1083.33 ± 144.33 Ω, corresponding to pH values between 7.02 and 3.5. Thereafter, it slowly increased to reach 1301.28 ± 178.68 Ω at the end of the acidification phase (T_390min_) when, in parallel, the pH continued to decrease to 3. In comparison, in EDBM-AEM, the global resistance decreased rapidly from 288.22 ± 39.69 Ω to 196.73 ± 18.22 Ω (T_60min_). Furthermore, in EDBM-MCP, after the addition of pepsin, the global resistance decreased slightly to a value of 849.2 ± 143.51 Ω, followed by a slight increase to 1086.3 ± 156.78 Ω after 3 h of hydrolysis (T_570min_) (Figure 4a) while in EDBM-AEM, it was constant at a value of 198.29 ± 37.79 Ω (T_240min_) (Figure 4b). The increase in global resistance observed for the cationic configuration was explained by the conductivity decrease in the acidified compartment due to the migration of free cations, as observed by Lin Teng Shee et al. [38]. Furthermore, as described in several studies, the ionic equilibrium is important during an electrodialytic process since an important impoverishment of one compartment in ion (low ash content of the hemoglobin solution) would increase the global system resistance and consequently will affect the current efficiency [37,40]. However, such a high increase in global resistance could not be fully explained by just the demineralization, and it is possible that the integrity of the MCP was changed or fouling was present on this membrane. For EDBM-AEM, the decrease in overall resistance was explained by an increase in conductivity of the acidified compartment due to migration of Cl^−^ anions from the KCl basified solution. In order to confirm or not the possible change in MCP integrity or potential fouling, the following section is dedicated to the characterization of the membranes after EDBM in terms of membrane thickness and electrical conductivity, as well as presenting photographs.

#### 3.2.4. Membrane Characterization 

Membranes were first characterized by measuring their thickness (cm) and conductance (Ohm) before and after each treatment as well as the conductance of the NaCl solution (0.5M), then the conductivity of the membrane (mS/cm) was calculated and presented in Table 2. In EDBM-MCP, a configuration formed by stacking one AEM, two MCPs and two BMs, statistical analysis demonstrated that conductivity of AEM did not significantly change before and after the treatment, with an averaged value of 5.3 ± 0.0 mS/cm (*p* > 0.05). However, statistical analyses demonstrated that conductivity of MCP-1, MCP-2, BM-1 and BM-2 significantly changed before and after the treatment (*p* < 0.05 for all membrane types). Concerning MCPs, a large variation in conductivity from an averaged value of 4.26 ± 0.03 before and 0.96 ± 0.10 mS/cm after the treatment was observed, while it was quite negligible for BM. In comparison, in EDBM-AEM, formed of three AEMs and two BMs, statistical analyses demonstrated that conductivity of AEM-1, AEM-2 and AEM-3 did not significantly change before and after the treatment (*p* > 0.05). However, conductivity values of BM-1 and BM-2 significantly changed before and after the treatment (*p* < 0.05). These data confirmed the absence of integrity change on AEM membranes and changes or presence of fouling on MCP membranes. The slight differences in conductivity observed for BM membranes could be due to a change in ash content after EDBM and/or to peptide precipitation on BM membranes based on electrostatic or hydrophobic interactions, as proposed by Kravtsov et al. [41] on whey deacidification. In addition, the highest difference in electrical conductivity observed for BMs in EDBM-MCP in combination with the decrease in conductivity of MCP can probably explain the difficulty of reducing and maintaining the pH and consequently the longer acidification duration. 

**Figure 5:**
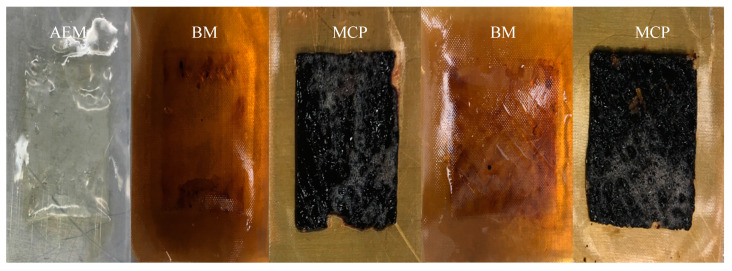
Hemoglobin fouling on EDBM-MCP membranes at the end of the treatment.

**Figure 6:**
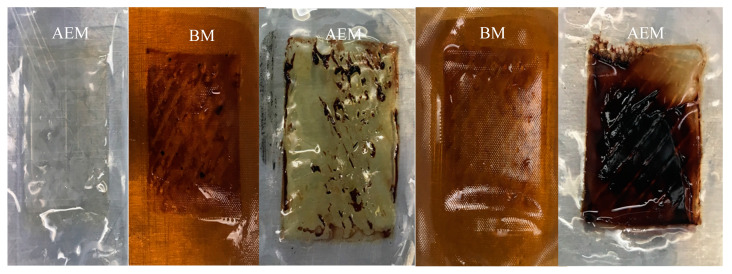
Hemoglobin fouling on EDBM-AEM membranes at the end of the treatment.

Figure 5 shows that, in the EDBM-MCP, the MCPs exhibited fouling, while bipolar and anionic membranes did not present any fouling. This confirmed the previous hypothesis concerning the integrity change of the MCP. In comparison, Figure 6 shows that, in EDBM-AEM, the anionic and bipolar membrane exhibited lower or even no fouling. Only the last anionic membrane had a fine deposit that may be due to hem or high protein concentration, but this did not affect the course of treatment.

The pH of the hydrolysate solution was close to 3.0: this allows electrostatic interactions between peptides and the MCP which may be responsible for its fouling. Indeed, at pH 3, the peptides were mainly positively charged and since the sulfonic groups of the MCP are negatively charged (SO_3_^−^), electrostatic bonds were formed, leading to peptide fouling, as described for conventional cation-exchange membrane by Persico et Bazinet [22]. However, the presence of a thin layer oppositely charged on the surface of the MCP matrix, to render it selective to monovalent cations, should have avoided the possibility of electrostatic interactions between peptides and the membrane surface to avoid or limit peptide fouling. It appeared that this thin layer repulsed the main part of positively charged peptides with high molecular weights, due to the restriction of the cavity size and/or highly charged positively peptides, due to the electrostatic repulsion of the thin layer, but some were able to interact with the matrix underneath. Indeed, Persico and Bazinet [22] observed that the presence of a positively charged layer at the interfaces of the same MCP as the one used in these experiments decreased drastically peptide fouling by 95–100% but did not completely avoid it. They also demonstrated with rhodamine B, a positively charged fluorescent compound, that rhodamine B was only detected in scattered cavities (probably crosslinking polymers) of MCP. However, this was not consistent with (1) a potential decrease in the peptide fouling expected since fouling on the MCP was major, (2) the dark red color of the fouling and (3) the fact that after hydrolysis, soaking fouled membranes in NaCl solution, as proposed by Persico et al. [36] to eliminate peptide fouling on ion-exchange membranes, was not enough to remove the entire red layer. So, this fouling was probably composed not only of peptides but also of hem, a molecule tightly attached to hemoglobin at neutral pH and responsible for its red color. Hence, according to the pH of the solution and the release of hem alone or protein fragments containing hem and peptides during hydrolysis, peptides may have first interacted electrostatically (positive residues) into the scattered cavities of the thin layer, where the negatively fixed group of the MCP matrix was reachable and, afterwards, hem or the protein fragments containing hem interacted with these already adsorbed peptides via hydrophobic interactions. Persico et al. [36] developed this hypothesis for a conventional cationic membrane (CMX-SB) when they observed that at pH 6, after fouling, four sequences of LIVTQTMK, ALPMHIR, TKIPAVFK and IPAVFK were present in both salt (decrease electrostatic interaction) and SDS solutions (competition with hydrophobic interactions). They demonstrated that a first layer was formed by positively charged peptide reacting by electrostatic interactions and a second one due to hydrophobic interaction through their respective hydrophobic residues. 

Earlier ESI-MS experiments sought to gain insights into the HB assembly and disassembly mechanism by studying the protein at increasing acid concentrations. Simmons et al. [42] and Boys et al. [43] suggested major differences in the behavior of alpha and beta-globin during pH denaturation. They studied the gradual changes in HB from pH 6.8 to 2.1 in a manner that can be described by a stepwise sequential unfolding mechanism inducing the separation of α chain, β chain and hem. Based on both structural considerations and experimental results, Lebrun et al. [44] highlighted the predominant role of the hydrophobic interactions in the formation of the hem–peptide complexes at acidic pH after denaturation, for which hem alone is completely insoluble. Nonetheless, this explanation could be proposed in our study: those positive peptides may have interacted firstly electrostatically with the negative sulfonic groups of the membranes and secondly through peptide–hem associations. Furthermore, it is important to mention that demineralization in EDBM-MCP could have also increased fouling. Decreasing in overall conductivity and low ionic strength may probably increase the electrostatic interaction of the peptide–membrane bonds due to a lack of potassium which could interact electrostatically with the membrane and avoid another peptide interaction. 

On the other hand, absence of or low fouling in EDBM-AEM (Figure 6) is related to the pH of the solution. The lower the pH, the more positively charged the peptides and, consequently, the weaker the ability of peptides to establish electrostatic interactions with the AEM (positively charged) leading to less fouling. The positively charged functional groups of the anionic membrane lead to electrostatic repulsions with peptides (positively charged). This explains the absence of or low fouling on the anionic membranes [35,45]. However, very thin red fouling was observed on the latest AEM, which may be due to possible hydroxyl ion leakage, which causes precipitation of hem or peptide–hem associations, as described for chitosan precipitation at the interface of the anionic membranes by Lin Teng Shee et al. [46]. 

### 3.3. Ash Content and Mineral Composition

#### 3.3.1. Evolution of Ash Content

**Figure 7:**
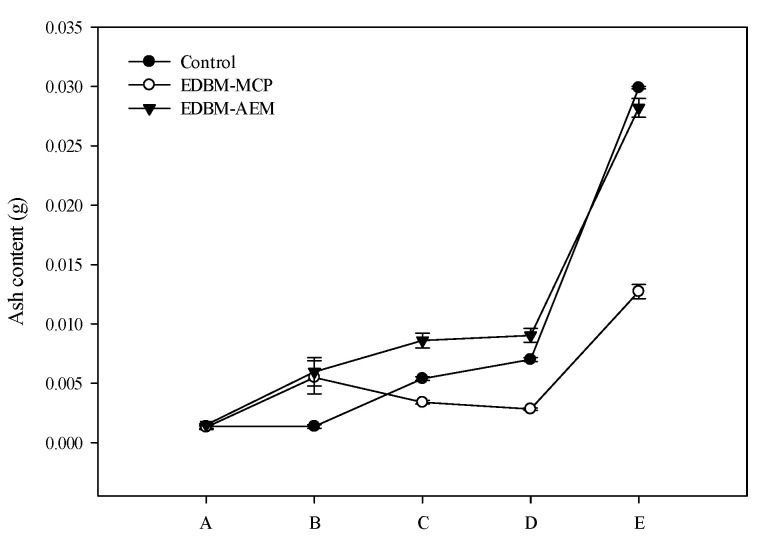
Ash content (g) evolution during three different processes. Letters A to E refer respectively to hemoglobin solution, conductivity adjustment (only for EDBM-MCP and EDBM-AEM), end of acidification, end of hydrolysis and reaction stop.

The ash content evolution, from the 1% initial HB solution to the end of the three processes (conventional hydrolysis (control), EDBM-MCP and EDBM-AEM), is shown in Figure 7. 

For 1% HB solution, at the beginning of the treatment, as expected, the ash content was the same whatever the conditions, with an average value of 0.0015 ± 0.0000 g. After conductivity adjustment for EDBM-MCP and EDBM-AEM, which corresponds to the addition of potassium chloride to ensure a minimum conductivity of the hemoglobin solution before its treatment in ED, as expected, the ash content increased. Since the same quantity of KCl was added (0.18 g) for both ED treatments, the final amount of ash reached an averaged value of 0.0057 ± 0.0002 g. Since no adjustment was carried out for conventional hydrolysis, the amount of ash remained the same. At the end of acidification, the ash content was significantly different (*p* < 0.05) between the three conditions. It increased to 0.0054 ± 0.0001 and 0.0086 ± 0.0006 g for control and EDBM-AEM, respectively, while it decreased to 0.0034 ± 0.0001 g in EDBM-MCP. At the end of the hydrolysis phase, the ash content was also significantly different (*p* < 0.05) between the three conditions. It continued to increase for control and EDBM-AEM to reach, respectively, 0.007 ± 0.0001 and 0.009 ± 0.0005 g while it still decreased for EDBM-MCP to reach 0.0028 ± 0.0001 g. After the reaction was stopped, the final ash content was significantly different (*p* < 0.05) between the three conditions. For all three processes, the amount of ash logically increased due to the addition of KOH (0.5 M) to stop the reaction at pH 9, but the final ash content was different between the three conditions. The highest amount of ash was reported for the control, with a final ash content of 0.0291 ± 0.0005 g in comparison to 0.0282 ± 0.0004 and 0.0127 ± 0.0001 g for EDBM-AEM and EDBM-MCP, respectively. During the hydrolysis phase in EDBM-MCP, the pH was at 3.67 ± 0.03, while it was maintained at 3 in control and EDBM-AEM, hence the need for a lower volume of KOH to adjust the pH at 9 in EDBM-MCP.

#### 3.3.2. Mineral Composition of the 1% HB Solution after Hydrolysis

The determination of the mineral composition in the hemoglobin solution at the end of hydrolysis under the three different conditions was important to test the feasibility of the ED configuration to reduce the salt concentration in comparison with conventional hydrolysis. The minerals identified are chloride, potassium, sulfur and sodium. The concentrations of these minerals (mg/L) are shown in Table 3.

Chloride concentration (mg/L) of hemoglobin solution after 3 h of hydrolysis (before stopping the reaction with KOH) was significantly different (*p* < 0.05) between control, EDBM-MCP and EDBM-AEM (Table 3). The higher chloride concentration observed in the control in comparison with EDBM conditions was due to the addition of hydrochloric acid (HCl) throughout the process to adjust and maintain the pH at 3. In EDBM, the pH was adjusted and maintained by the bipolar membrane without addition of chemicals. Although KCl was added, for EDBM experiments, to adjust the conductivity of the solution, the final concentration was lower than the one of the control. Furthermore, the lower Cl^−^ ion concentration in EDBM-MCP was due to the migration through the AEM of Cl^−^ ions from the basified KCl solution to the hemoglobin compartment to keep it electroneutral, while the MCP did not allow the migration of Cl^−^ ions, so they remained in the hemoglobin compartment, as discussed previously (Section 3.2.2). 

Potassium concentration (mg/L) of hemoglobin solution was significantly different (*p* < 0.05) for control vs. EDBM-AEM and EDBM-MCP vs. EDBM-AEM, but no difference was observed between control and EDBM-MCP. The higher K^+^ concentration observed in EDBM-AEM was due to the fact that K^+^ were retained in hemoglobin compartment by the AEM, while K^+^ migrated through the MCP, as explained previously (Section 3.2.1). For the control, its low concentration in potassium is related to its low concentration in the hemoglobin powder since no potassium was added throughout this treatment. 

Sulfur concentration (mg/L) significantly changed (*p* < 0.05) between the three treatments. The higher sulfur content in EDBM-AEM than in control and EDBM-MCP was due to the migration of sulfate ions (SO_4_^2−^) from the electrode rinsing solution (Na_2_SO_4_) through the AEM. For control and EDBM-MCP, since, throughout the treatment, there was no addition of sulfur, the low concentration of sulfur observed is probably due to its presence in the starting hemoglobin powder. 

Finally, sodium concentration (mg/L) significantly changed (*p* < 0.05) for control vs. EDBM-AEM and EDBM-MCP vs. EDBM-AEM, and no significant difference appeared between control and EDBM-MCP. Since no addition of sodium was carried out, the concentration of sodium observed is probably due to its presence in the starting hemoglobin powder for the three conditions. 

### 3.4. Enzymatic Kinetics and Mechanism of Action of the New Process

#### 3.4.1. Determination of the Degree of Hydrolysis

**Figure 8:**
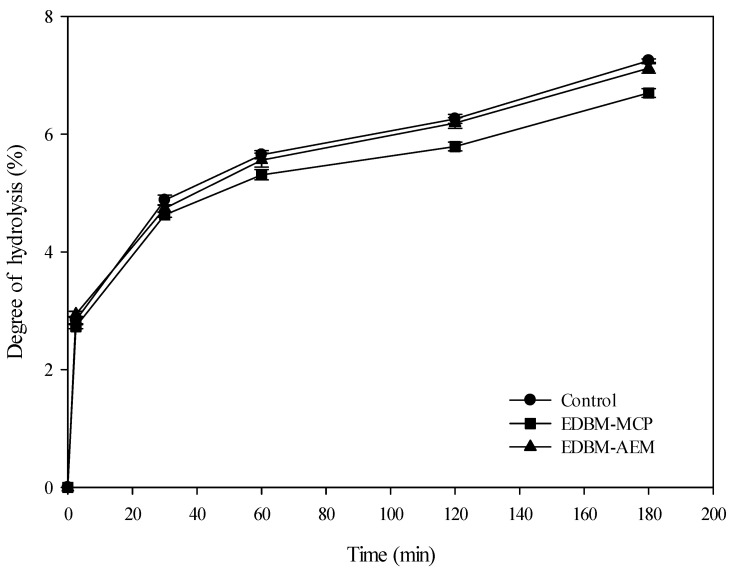
Hydrolysis curves of hemoglobin hydrolysis in control, EDBM-MCP and EDBM-AEM treated with pepsin for 3 h. Hydrolysis was conducted at the following conditions: enzyme (U)/protein (mg) ration of 1/11, pH 3 and at 30 °C.

The degree of hydrolysis (DH) has an identical tendency to increase over time in control, EDBM-MCP and EDBM-AEM (Figure 8). The shape of the curves was identical to those previously published in the literature [12]. For control and EDBM-AEM, the DH evolution was identical, while a statistical difference was observed (*p* < 0.05) between the control and EDBM-MCP. A higher DH was observed for the control compared to EDBM-MCP and EDBM-AEM. Hydrolysis of bovine hemoglobin was characterized by a high rate of hydrolysis during the initial 30 min for the three conditions. At T_60min_, the rate of enzymatic hydrolysis increased slightly, and then it continued to increase quite linearly until 180 min. At 180 min, the degree of hydrolysis was not significantly different (*p* > 0.05) between control (7.25 ± 0.03) and EDBM-AEM (7.12 ± 0.08), while there was a significant difference (*p* < 0.05) between control (7.25 ± 0.03) and EDBM-MCP (6.7 ± 0.076) as well as between EDBM-MCP (6.7 ± 0.076) and EDBM-AEM (7.12 ± 0.08). The lower DH was observed in EDBM-MCP, meaning that there was a slow-down in enzymatic kinetics during the hydrolysis of hemoglobin in this special condition. This lower final DH could be due to the fouling of MCP containing peptides that were not hydrolyzed and/or to the inability to adjust the pH to 3 during the hydrolysis phase, affecting consequently the efficiency of pepsin. Indeed, it was demonstrated that pH 3 was the most efficient pH for hemoglobin hydrolysis by pepsin while keeping the solution homogeneous [4]. 

#### 3.4.2. Reaction Mechanism of Bovine Hemoglobin Hydrolysis by Pepsin and Identification of α137-141 Peptide 

To understand the different enzymatic kinetics and to identify the enzymatic mechanism of bovine hemoglobin hydrolysis obtained in EDBM and control, the UPLC-QTOF chromatographic profiles obtained as a function of time for the three conditions were analyzed. Chromatographic profiles of hydrolysis in control, EDBM-MCP and EDBM-AEM are shown, respectively, in the Supplementary Materials, in Figures S2, S3 and S4. The most striking fact was that the chromatograms of control, EDBM-MCP and EDBM-AEM were almost the same. 

Three peaks are shown at T_0_ before adding the pepsin: α-chain, β-chain and hem (see Supplementary Materials, Figures S2, S3 and S4). After adding pepsin, the hemoglobin subunits were rapidly hydrolyzed to intermediate peptides which were converted to final peptides, smaller and generally more hydrophilic than intermediate peptides. This enzymatic mechanism, called “Zipper”, was described for the first time by Linderstrom-Lang [47]. The enzymatic hydrolysis of bovine hemoglobin in EDBM-MCP and EDBM-AEM allowed a “Zipper” mechanism identical to the one obtained in conventional hydrolysis (control). When hemoglobin is completely denatured by adjusting the pH to 3, it allows the complete opening of its globular chain. This privileges the enzymatic mechanism “Zipper” at the expense of the “one-by-one” mechanism, observed for higher pH [48]. This mechanism allowed us to obtain α137-141 peptide that was identified by UPLC-MS/MS. The same mechanism was observed in other studies for conventional hydrolysis of hemoglobin and described by Nedjar-Arroume et al. [10], Nedjar-Arroume et al. [15] and Dubois et al. [48]. α137-141 appeared identically in the three conditions from the beginning of hydrolysis and its concentration increased throughout the course of proteolysis. The α137-141 production was fast—it appeared after only a few minutes from the start of hydrolysis. It thus accumulated during hydrolysis, meaning that it was a final peptide, not being hydrolyzed in other shorter peptides under the conditions of hydrolysis used here (30 °C; pH 3, C_BH_ of 1% w/v). This confirmed what was previously described by Lignot et al. [4]. Moreover, these conditions have shown an important accumulation of α137-141 peptide, explained by the “Zipper” mechanism. The peptide α137-141 represents the last five amino acids at the C-terminal of the α chain of bovine hemoglobin, namely Thr-Ser-Lys-Tyr-Arg (TSKYR), which has a molar mass of 653 Da and has an isoelectric point of 10.5. It is thus charged +2 at a pH of 7 [24]. 

#### 3.4.3. Characterization of the Peptide Populations 

##### Principal Component Analysis (PCA) and Heat Maps

**Figure 9:**
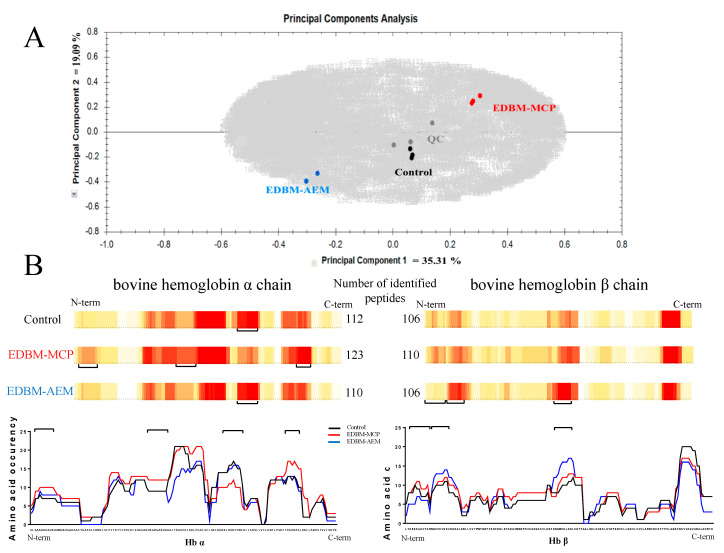
(**A**) Principal component analysis based on comparison of mass spectrometry detected ions in bovine hemoglobin hydrolysates. Quality control (QC) samples correspond to equivolume mixtures of the 9 hydrolysates (three control, three EDBP-AEM and three EDBM-MCP) injected at the beginning, the middle and the end of the HPLC-MS/MS analysis session. (**B**) Heat maps showing amino acid occurrence in the identified peptides in the primary sequences of the bovine hemoglobin α and β chains after its hydrolysis in the three conditions. Underlined regions highlight peptide pattern differences. Heat maps were generated after combination of three LC-MS/MS analysis for each replicate.

In order to investigate the different hydrolysis conditions, hydrolysates obtained following 3 h of hydrolysis were selected for peptide identification by LC-MS/MS. PCA was performed on the mass spectrometry data after importation of raw data in Progenesis QI for proteomics software (version 4.1). Peptic maps, representing retention time of the ions according to the m/z ratio of the ions and the intensity of the ions, obtained for all hydrolysates, were automatically and manually aligned thanks to the quality control used and peak picking of mass signals was constrained (Section 2). The PCA was done using the detected peptides and showed the peptide population obtained by considering all the mass signals (Figure 9A). The first two dimensions in the PCA explained 54.4% of the variance (detected ions corresponding to variables). The more distant the groups were, the more different they were in terms of ion population. The PCA clearly showed a similar ion population and thus a similar peptide population between the three repetitions of each condition, meaning that it was reproducible at the condition level. Additionally, PCA showed a slight difference in ion population and thus a slight difference in peptide population between control, EDBM-MCP and EDBM-AEM. Moreover, the divergence between the different identified peptides in control, EDBM-MCP and EDBM-AEM were subsequently presented in the form of a heat map via Peaks Studio Software (Version 8.5, Bioinformatics Solutions Inc., Waterloo, ON, Canada)) (Figure 9B). 

For each amino acid from the protein sequence, a score was calculated according to its occurrence in each peptide sequence identified. The higher the occurrence, the more the peptide zone will tend towards red. The red areas represent the areas where amino acids are identified very often, the lighter areas where amino acids are poorly identified and the white areas where there has been no identification. This makes it easy to see the areas which are resistant to hydrolysis (red areas). For the three conditions, some color similarities were observed in certain parts of the map as well as differences (underlined regions in Figure 9). 

The number of identified peptides in the alpha chain of hemoglobin between the control and EDBM-AEM was roughly the same, with 112 and 110 peptides, respectively, while 123 peptides were identified in EDBM-MCP. The numbers of identified peptides for bovine hemoglobin β chain were 106, 110 and 106 for the control, EDBM-MCP and EDBM-AEM, respectively. A slightly higher number of identified peptides was identified in EDBM-MCP, for bovine hemoglobin α and β chains, in comparison with control and EDBM-AEM. This was probably due to the presence of precursors in EDBM-MCP which were not hydrolyzed in other shorter peptides because of a slow-down in the kinetics of enzymes. This finding confirmed the results observed for degree of hydrolysis. Moreover, this difference can also be explained by the fact that the three conditions were tested with different acidification times, different types of membranes and different mineral contents. 

#### 3.4.4. Quantification of α137-141 Derived from the New Process

Since α137-141 was identified in the three conditions, its quantification was done by RP-HPLC. This measurement was made for C_BH_ = 1% (w/v), with ratio enzyme/substrate = 1/11 (mole/mole) after three hours of hydrolysis for control, EDBM-MCP and EDBM-AEM. For control, C_α137-141_ was 1.11 ± 0.16 μg/mL, while it was 1.13 ± 0.05 μg/mL in EDBM-MCP and 1.09 ± 0.09 μg/mL in EDBM-AEM. Statistical analysis demonstrated that C_α137-141_ was the same (*p* > 0.05) for the three conditions. Therefore, the bipolar membranes made it possible to produce the same amount of α137-141 peptide as in the control, without the use of chemical agents.

## 4. Conclusions

In this study, hydrolysis by EDBM was investigated to obtain the α137-141 peptide, a peptide of great interest for the food industry since it is considered as a natural antimicrobial and antioxydant peptide. It appeared from these results that EDBM was efficient to denature hemoglobin by reducing the pH to 3 and then to regulate the pH of the solution during hydrolysis. Furthermore, it was demonstrated that hydrolysis in EDBM has the same enzymatic mechanism as the conventional hydrolysis, the “Zipper” mechanism. This mechanism made it possible to obtain a large peptide population that was approximately the same as in the control and without the use of chemical solvents. This new successful concept, based on the generation of protons from water dissociation to control hydrolysis, also allowed the production of α137-141 at the same concentration as conventional hydrolysis. Hence, EDBM-MCP, allowed simultaneous demineralization of the hemoglobin solution compared to conventional hydrolysis and EDBM-AEM and production of α137-141 peptide with a low mineral content. In comparison, EDBM-AEM allowed the production of α137-141 peptide more rapidly without membrane fouling and involving minimal global resistance and consequently a lower energy consumption.

In this study, electrodialysis with bipolar membrane (EDBM) was demonstrated for the first time as an ecoefficient and innovative technology to produce peptide hydrolysates from enzymatic hydrolysis of bovine hemoglobin with low mineral salt concentration. This was due to the specific characteristics of the bipolar membranes which allow us to generate in situ H^+^ and OH^−^ ions from water dissociation under an electrical field and consequently do not need the use of any chemical agents. Further experiments will focus on minimizing peptide fouling on the monovalent permselective membrane, which can be an effective means of improving bovine hemoglobin demineralization, by applying a pulsed electric field or an overlimiting current regime, by changing the configuration of EDBM or by modification of ion-exchange membranes [49].

This new green application of EDBM to the production of α137-141 from a by-product of slaughterhouses fits perfectly with the concept of circular economy. Indeed, hemoglobin from blood, once hydrolyzed, allows the bioproduction of active peptides that can then be used in meat or meat products to increase their preservation and inocuity. 

## Figures and Tables

**Table 1:** Hemoglobin composition (%w/w).

Protein (%)	≃98
Ash (%)	≤2
Water (%)	≤8
Iron (Fe) (%)	0.25–0.35

**Table 2:** Conductivity (mS/cm) of MCP (monovalent cation perm-selective membrane), AEM (anion exchange membrane) and BM (bipolar membrane) before and after treatment in EDBM-MCP and EDBM-AEM.

Process	EDBM-MCP					EDBM-AEM				
Conductivity (mS/cm)	AEM	MCP-1	MCP-2	BM-1	BM-2	AEM-1	AEM-2	AEM-3	BM-1	BM-2
Before	5.3 ± 0.05 ^a^	4.3 ± 0.1 ^a^	4.23 ± 0.05 ^a^	5.8 ± 0.05 ^a^	5.7 ± 0.2 ^a^	5.3 ± 0.15 ^a^	5 ± 0.2 ^a^	4.9 ± 0.2 ^a^	5.9 ± 0.2 ^a^	5.8 ± 0.15 ^a^
After	5.3 ± 0.15 ^a^	0.86 ± 0.11 ^b^	1.06 ± 0.25 ^b^	5.06 ± 0.05 ^b^	5.13 ± 0.15 ^b^	5.1 ± 0.05 ^a^	4.76 ± 0.25 ^a^	5.06 ± 0.2 ^a^	5.33 ± 0.2 ^b^	5.4 ± 0.11 ^b^

Population means within each column with different letters are significantly different *p* < 0.05 (Tukey).

**Table 3:** Mineral concentration (mg/L) of hemoglobin solution at the end of the hydrolysis.

Conditions	Cl	K	S	Na
Control	388.3 ± 5.03 ^a^	3.73 ± 0.34 ^a^	61.35 ± 1.31 ^a^	2.85 ± 0.11 ^a^
EDBM-MCP	197.6 ± 4.04 ^b^	6.48 ± 0.15 ^a^	41.37 ± 0.98 ^b^	1.68 ± 0.8 ^a^
EDBM-AEM	235.6 ± 4.16 ^c^	364.76 ± 3.8 ^b^	313.69 ± 3.87 ^c^	23.35 ± 2.06 ^b^

P = probability level for the difference in mineral concentrations between control, EDBM-MCP and EDBM-AEM (*p* < 0.05); ^a–c^: Population means within each column with different letters are significantly different *p* < 0.05 (Tukey).

## Supplementary Materials

The following are available online at https://www.mdpi.com/2077-0375/10/10/257/s1, Figure S1: Diagram illustrating the acidification and hydrolysis of bovine hemoglobin in conventional hydrolysis (control) and by EDBM, Figures S2–S4: Chromatographic profiles of hydrolysis of bovine hemoglobin at 214 nm by UPLC-QTOF, analyzed by C18 column at different hydrolysis degrees for 3 h (pH 3, 30 °C, E/S = 1/11, CBH = 1%, w/v) in control, EDBM-MCP and EDBM-AEM, respectively.
