# Supplementary material for: Bovine Hemoglobin Enzymatic Hydrolysis by a New Ecoefficient Process—Part I: Feasibility of Electrodialysis with Bipolar Membrane and Production of Neokyotorphin (α137-141)
Source: Membranes (Basel). 2020 Sep 25;10(10):257. doi: 10.3390/membranes10100257 (PMC7600281; doi:10.3390/membranes10100257)
Supplement: Supplementary file 1 [file membranes-10-00257-s001.pdf]

membranes

Supplementary Materials: Bovine Hemoglobin Enzymatic Hydrolysis by a New Ecoefficient Process—Part I: Feasibility of Electrodialysis with Bipolar Membrane and Production of Neokyotorphin (α137-141)

Mira Abou-Diab [1,2,3,4], Jacinthe Thibodeau [1,2,3], Barbara Deracinois [4], Christophe Flahaut [4], Ismail Fliss [1,3], Pascal Dhulster [4], Naima Nedjar [4,*,†], and Laurent Bazinet [1,2,3,*,†]

[1] Department of Food Science, Université Laval, Québec, G1V 0A6, Canada; mira.abou-diab.1@ulaval.ca (M.A.D.); jacinthe.thibodeau.1@ulaval.ca (J.T.); ismail.fliss@fsaa.ulaval.ca (I.F.)
[2] Laboratory of Food Processing and Electromembrane Process (LTAPEM), Université Laval, Québec, G1V 0A6, Canada
[3] Institute of Nutrition and Functional Foods (INAF), Université Laval, Québec, G1V 0A6, Canada
[4] UMR Transfrontalière BioEcoAgro N°1158, Univ. Lille, INRAE, Univ. Liège, UPJV, YNCREA, Univ. Artois, Univ. Littoral Côte d'Opale, ICV - Institut Charles Viollette, F-59000 Lille, France; barbara.deracinois@univ-lille.fr (B.D.); christophe.flahaut@univ-artois.fr (C.F.); pascal.dhulster@univ-lille.fr (P.D.)
* Correspondence: naima.nedjar@univ-lille.fr (N.N.); Tel.: + 33 3 28 76 73 90 (N.N.); Fax.: + 33 3 28 76 73 56 (N.N.); Laurent.Bazinet@fsaa.ulaval.ca (L.B.); Tel.: +1-418-656-2131 (L.B.); Fax: +1-418-656-3353 (L.B.)
† These authors contributed equally to this work

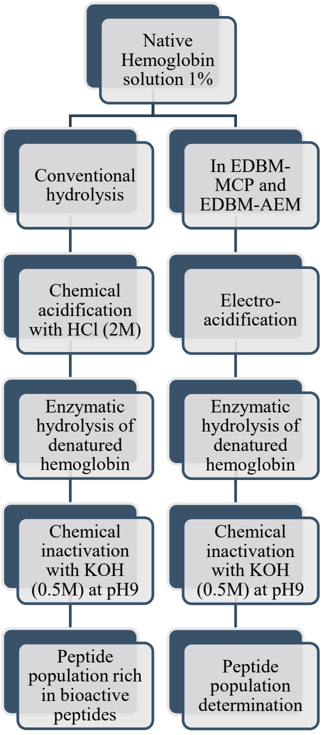

Figure S1. Diagram illustrating the acidification and hydrolysis of bovine hemoglobin in conventional hydrolysis (control) and by EDBM.

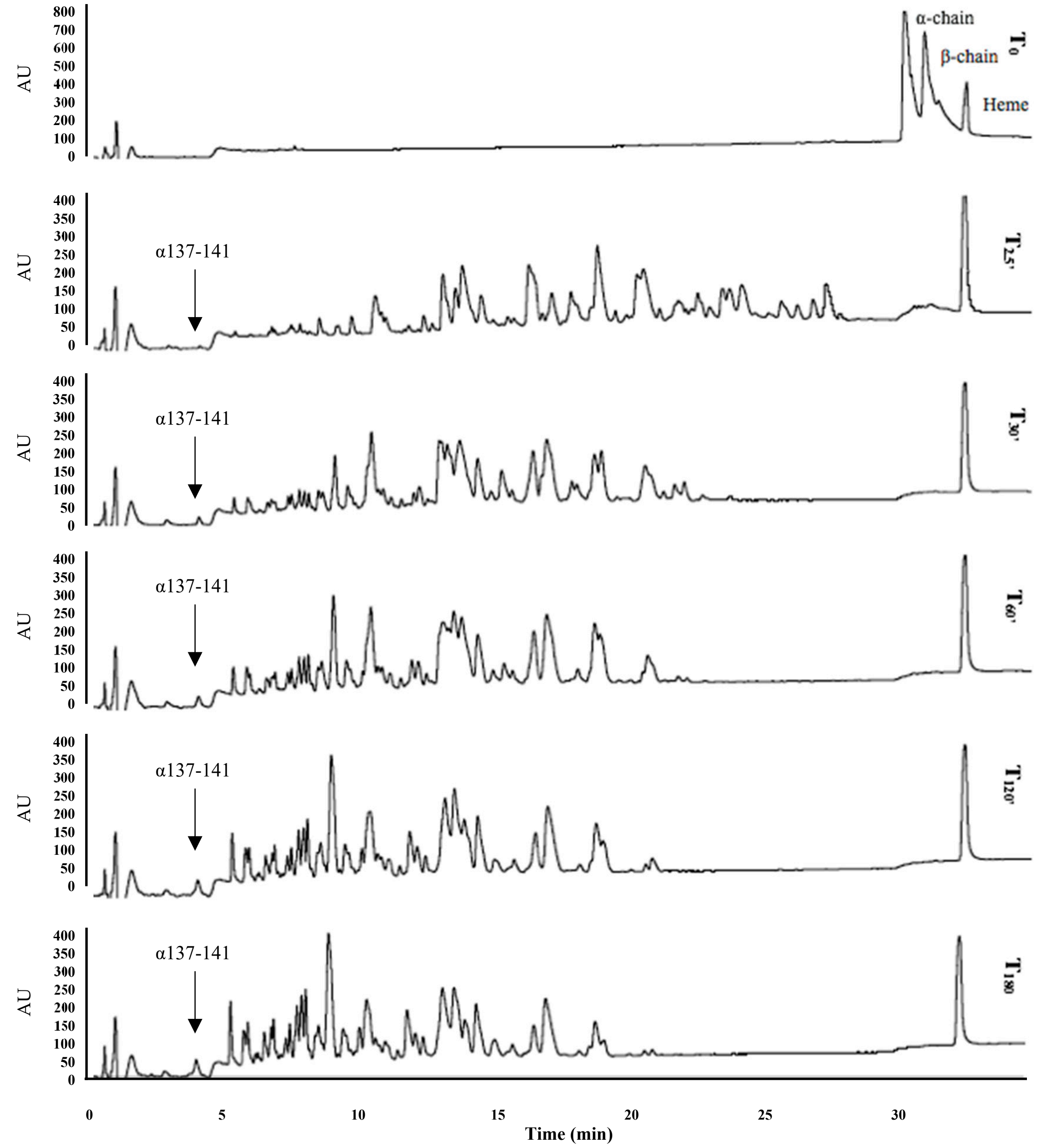

Figure S2. Chromatographic profiles of hydrolysis of bovine hemoglobin in control at 214 nm by UPLC-QTOF, analyzed by C18 column at different hydrolysis degrees for 3 hours (pH 3, 30°C, E/S = 1/11, CBH = 1%, w/v).

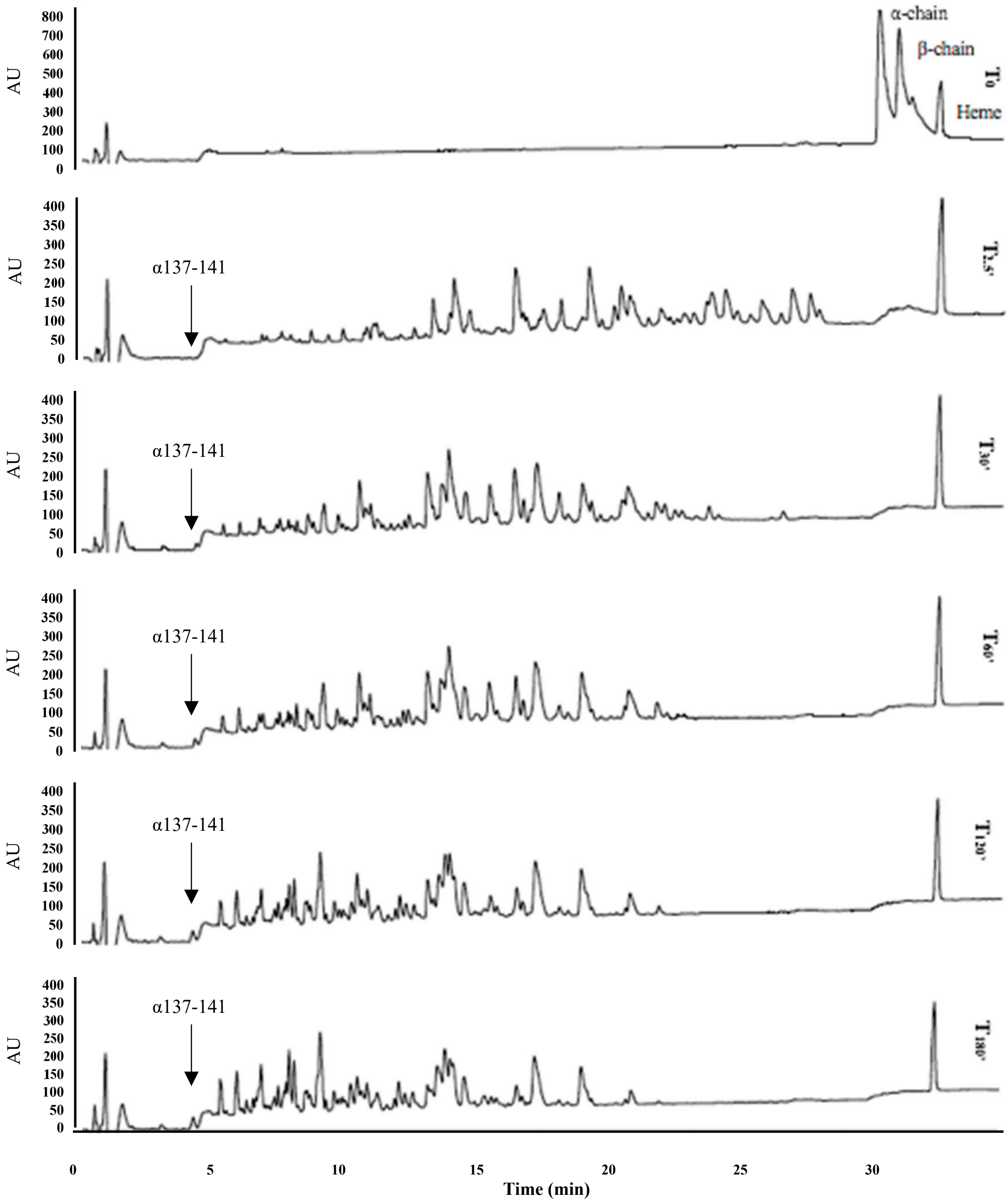

Figure S3. Chromatographic profiles of hydrolysis of bovine hemoglobin in EDBM-MCP at 214 nm by UPLC-QTOF, analyzed by C18 column at different hydrolysis degrees for 3 hours (pH 3, 30°C, E/S = 1/11, CBH = 1%, w/v).

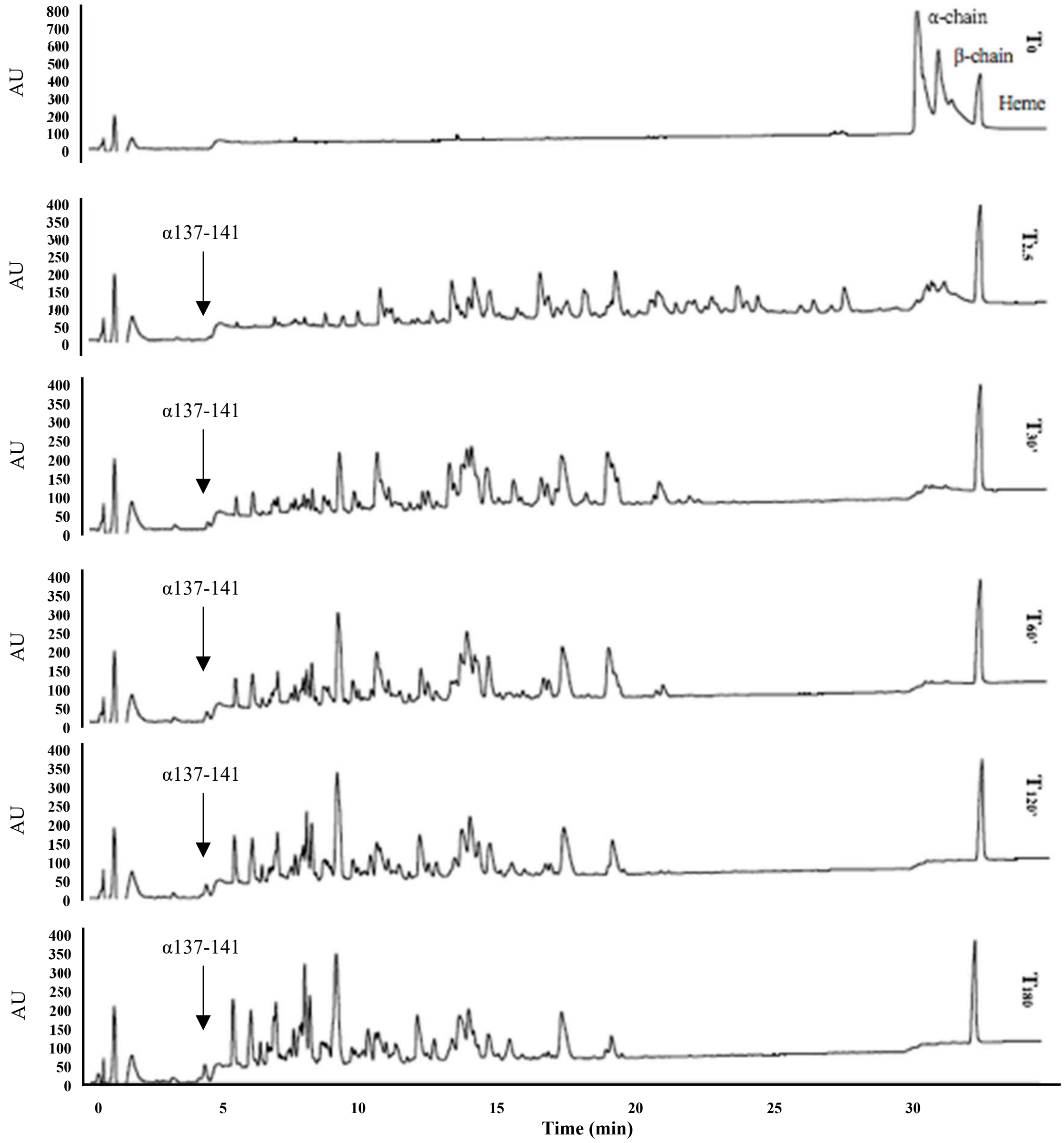

Figure S4. Chromatographic profiles of hydrolysis of bovine hemoglobin in EDBM-AEM at 214 nm by UPLC-QTOF, analyzed by C18 column at different hydrolysis degrees for 3 hours (pH 3, 30°C, E/S = 1/11, CBH = 1%, w/v).
